# Mathematical modeling of cholera dynamics with intrinsic growth considering constant interventions

**DOI:** 10.1038/s41598-024-55240-0

**Published:** 2024-02-26

**Authors:** Kewani Welay Brhane, Abdulaziz Garba Ahmad, Hina Hina, Homan Emadifar

**Affiliations:** 1 Department of Mathematics, Mekelle University, Mekelle, Tigray Ethiopia; 2 Department of Applied Mathematics, Federal University of Technology, Babura, Jigawa State Nigeria; 3 Department of Mathematics and Statistics, Women University Swabi, Swabi, KP Pakistan; 4 Department of Mathematics, Saveetha School of Engineering, Saveetha Institute of Medical and Technical Sciences, Saveetha University, Chennai, 602 105 Tamil Nadu India; 5 MEU Research Unit, Middle East University, Amman, Jordan; 6 Department of Mathematics, Hamedan Branch, Islamic Azad University of Hamedan, Hamadan, Iran

**Keywords:** Basic reproduction number, Cholera dynamics, Intervention rates, Mathematical modeling, Stability analysis, Medical research, Mathematics and computing

## Abstract

A mathematical model that describes the dynamics of bacterium vibrio cholera within a fixed population considering intrinsic bacteria growth, therapeutic treatment, sanitation and vaccination rates is developed. The developed mathematical model is validated against real cholera data. A sensitivity analysis of some of the model parameters is also conducted. The intervention rates are found to be very important parameters in reducing the values of the basic reproduction number. The existence and stability of equilibrium solutions to the mathematical model are also carried out using analytical methods. The effect of some model parameters on the stability of equilibrium solutions, number of infected individuals, number of susceptible individuals and bacteria density is rigorously analyzed. One very important finding of this research work is that keeping the vaccination rate fixed and varying the treatment and sanitation rates provide a rapid decline of infection. The fourth order Runge–Kutta numerical scheme is implemented in MATLAB to generate the numerical solutions.

## Introduction

The infectious disease cholera, which is an acute intestinal illness, continues to pose a serious risk to public health in nations with limited resources. From its initial reservoir in the nineteenth-century Ganges delta in India, it expanded around the world. An estimated 1.3–4.0 million cases of cholera and 21,000–143,000 fatalities globally are attributed to the disease each year^1–12^. The dynamics of the infection involve multiple interactions between the human host, the pathogen, and the environment, which contribute to both human to human and environment to human transmission pathways^1–11^. The most common pathways of contracting the germs are by eating food cooked by infected people, drinking contaminated water, and shaking hands with infected people. The main symptom of cholera is severe acute watery diarrhoea that lasts for three to seven days. If treatment is delayed, this can lead to excessive and fast dehydration and possibly death^1–12,14–20^.

In the effort of fighting against cholera, multifaceted approach, such as surveillance, water, sanitation, and hygiene (WaSH) measures, social mobilization, treatment, and oral cholera vaccines are have been implementing. A worldwide plan for cholera control, “Ending Cholera: a global roadmap to 2030,” was introduced in 2017 with the goal of lowering cholera mortality by 90%^13,21^. In the developed countries, cholera has been effectively eliminated through investments in proper environmental health solutions, such as water supply, treatment, sanitation infrastructure, and hygiene-related behavior change^13,21^. However, cholera is still challenging the developing countries due to factors such as poor water supplies, worsening sanitation, socioeconomic conditions, climate change, and humanitarian crises. In Africa, cholera has persisted due to worsening hygiene and sanitation in urban areas, with the burden likely to worsen without measures to improve water and hygiene infrastructure^21–24^. The World Health Organization (WHO) reports that drivers for current cholera outbreaks include widespread floods, droughts, humanitarian crises, political instability, and conflict, all of which are more prevalent in developing countries^23,24^.

For an extended period, mathematical modelling has yielded valuable insights for a more profound comprehension of the intricate dynamics of cholera. In an effort to understand the fundamentals of the spread of cholera and to quantify effective control strategies, a large number of mathematical models for the dynamics of bacterial spreading have been created. To more properly depict the pattern of the disease infection spread, a few of these models included two types of infection routes^1–11,14,16,25^. However, a significant drawback of the modelling studies that are currently being conducted on the transmission of cholera is the lack of attention given to the intrinsic dynamics of the bacteria, which results in an inadequate comprehension of the development of the bacteria and how it affects the dynamics of infection. The majority of mathematical models of cholera often make the premise that bacteria cannot survive without human assistance. In^2–11,17,18,20,25,28,30^, mathematical models that do not consider bacterial intrinsic growth are presented. This is predicated on a cholera ecological early hypothesis documented in^27^. A straightforward depiction of the rate of change for the bacterial density is made possible by the assumption. Regretfully, new ecological studies have provided compelling evidence that the bacteria may reproduce and thrive on their own in a variety of aquatic habitats. These ecological findings require more modelling effort to better understand the internal dynamics of cholera diseases and the connection between environmental persistence and disease outbreaks^16^. In^14,16^, efforts are done to develop and analyze mathematical models considering bacterial intrinsic growth. However, the main gap in the existing mathematical models of cholera are there is no mathematical model that incorporated both bacterial intrinsic growth and intervention strategies. This gap is also available in the recent publications by^14,16^.

Our goal in this work is to develop and analyze a mathematical model that incorporates both bacterial intrinsic growth and intervention strategies with an intention to fill the gap in^14,16^. Such kind of mathematical model will provide valuable advice for efficient preventive and intervention techniques against cholera outbreaks. The current mathematical model examines cholera dynamics using intrinsic bacterial growth rate and control measures that are integrated into the mathematical model of^14,16,25,28^ in order to achieve this goal. In addition to the inherent bacteria growth rate, we modify the existing models^1–11,14,16,25^ by including three controlling mechanisms, Namely; immunization, therapeutic treatment, and water sanitation. Rigorous mathematical theories are applied to examine the impacts of intrinsic bacteria growth rate, various control measures, and several cholera transmission channels^26^.

This article contains seven sections and is organized as follows. A detailed introduction to cholera modeling is presented in section "Mathematical model formulation". In section "Mathematical model formulation", model assumptions are presented and a corresponding mathematical model is formulated. The positivity of the domain of biological interest is also analyzed in this Section. The mathematical model is validated against a WHO real cholera data in section "Model validation". In section "Mathematical analysis of equilibrium solutions", is rigorously presented. The existence and stability of epidemic and endemic equilibrium solutions are examined in this Section. Section "Numerical test problems for stability of equilibrium solutions" provides information on how model parameters affect the presence and stability of equilibrium solutions. Furthermore, in section "Model parameter sensitivity analysis", sensitivity analysis of a few model parameters is given. Lastly, Section presents the conclusion and subsequent actions. Section "Conclusion and future works".

## Mathematical model formulation

We have taken into account the following hypotheses when creating the mathematical model.

1. Because the infection phase is brief, there is a low risk of death and births. This assumption leads us to classify the total human population (*N*) into susceptible number (*S*), infective number (*I*), and recovered number (*R*) so that
  1 $$\begin{aligned} N(t)=S(t)+I(t)+R(t), \quad \forall \, t\ge 0, \end{aligned}$$
  where *t* represents time.
2. Individuals are born susceptible.
3. The bacteria has an intrinsic rate of growth of *r* and a weight capacity of $\kappa$. Its concentration in the environment is always there, and we represent it as *B*.
4. The bacterium can spread from person to person, from environment to environment, and from human to environment at rates of $\alpha$, $\xi$, and $\beta$, respectively.
5. The susceptible population is vaccinated at a rate of *v*, resulting in the removal of *vS*(*t*) people from the susceptible class and their addition to the recovered class each time.
6. In order to remove *aI*(*t*) persons from the affected class and add them to the recovered class, therapeutic treatment is administered to those who have been infected at a rate of *a*.
7. A recovered individual is assumed to develop immunity.
8. Bacteria perish as a result of water sanitation at a rate of *w*.

**Figure 1:**
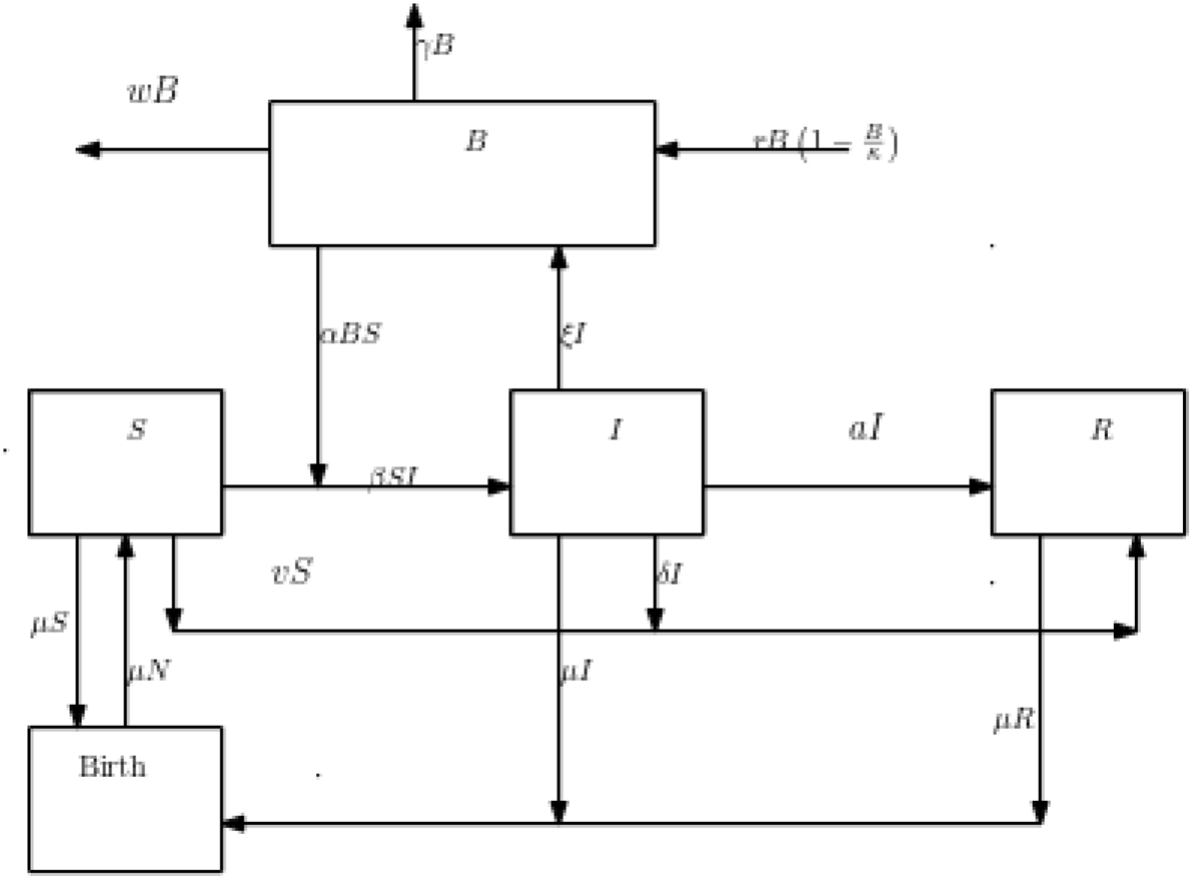
Flow diagram of the mathematical model.

Figure 1 provides a graphical depiction of the model.

Based on the previous supposition, we derive the subsequent dynamic system:

2 $$\begin{aligned} \frac{dS}{dt}= & {} \mu N-(\alpha I + \beta B)S-\mu S -vS, \end{aligned}$$

3 $$\begin{aligned} \frac{dI}{dt}= & {} (\alpha I + \beta B)S-\mu I- (\delta +a)I, \end{aligned}$$

4 $$\begin{aligned} \frac{dR}{dt}= & {} (\delta +a)I- \mu R +vS, \end{aligned}$$

5 $$\begin{aligned} \frac{dB}{dt}= & {} \xi I + r B\left( 1-\frac{B}{\kappa }\right) -\gamma B-wB. \end{aligned}$$

The rates of birth, infection recovery, natural death, and contribution from infected individuals to the environmental bacterial population are represented by the variables $\mu$, $\gamma$, and $\xi$ in Eqs. (2)–(5). Positive values are assumed for each of these parameters. To evaluate the following system of differential equations in our mathematical analysis, we will first utilise $R=N-I-S$ to remove Eq. (4).

6 $$\begin{aligned} \frac{dS}{dt}= & {} \mu N-(\alpha I + \beta B)S-\mu S -vS, \end{aligned}$$

7 $$\begin{aligned} \frac{dI}{dt}= & {} (\alpha I + \beta B)S-\mu I- (\delta +a)I, \end{aligned}$$

8 $$\begin{aligned} \frac{dB}{dt}= & {} \xi I + r B\left( 1-\frac{B}{\kappa }\right) -\gamma B-wB. \end{aligned}$$

### Lemma 1

For any time $t\ge 0$, all solutions of the dynamical system in Eqs. (2)–(5) with positive initial conditions are non-negative within the region of biological interest.

9 $$\begin{aligned} \Gamma =\left\{ \left( S,I,R,B\right) \in {\mathbb {R}}_+^4: S, I,R,B\ge 0,\, S+I+R=N \right\} . \end{aligned}$$

Additionally, $\Gamma$ exhibits positive invariance.

### Proof

Suppose that

10 $$\begin{aligned} \tau = \text {sup}\left\{ t\ge 0: S(t),I(t),R(t),B(t)>0\right\} . \end{aligned}$$

If $\tau =\infty$, then $S(\tau ), I(\tau ), R(\tau ), B(\tau )>0$ and the claim of Lemma 1 is satisfied. If $\tau$ is finite, then we have to go farther to prove the claim. Defining $\eta (t):=\alpha I(t)+ \beta B(t)+\mu +v$, Eq. (2) of the dynamical system can be written as

11 $$\begin{aligned} \frac{dS}{dt}+\eta S=\mu N. \end{aligned}$$

An expression for the aforementioned equation is

12 $$\begin{aligned} \frac{d}{dt}\left[ S(t)\exp \left\{ \int _{0}^{t}\eta (s)ds\right\} \right] =\mu N \exp \left\{ \int _{0}^{t}\eta (s)ds\right\} . \end{aligned}$$

The particular solution to Eq. (12) at $t=\tau$ is derived to be

13 $$\begin{aligned} S(\tau )=\exp \left\{ - \int _{0}^{t}\eta (s)ds\right\} \left[ S(0)+\mu N \int _{0}^{\tau }\left\{ \exp \left\{ \int _{0}^{t}\eta (s)ds\right\} \right\} dt\right] >0. \end{aligned}$$

Through the same procedure, it can be easily verified that $I(\tau ), R(\tau ), B(\tau )>0$. Now, adding Eqs. (2)–(4) , we have that

14 $$\begin{aligned} \frac{dS}{dt}+\frac{dI}{dt} + \frac{dR}{dt}=0. \end{aligned}$$

Integrating the above equation with respect to time , we have that

15 $$\begin{aligned} S(t)+I(t)+R(t)=c, \forall t, \end{aligned}$$

where *c* is a constant real number. Now, let $S(0), I(0), R(0) >0$ be initial values in $\Gamma$, then

16 $$\begin{aligned} S(t)+I(t)+R(t)=S(0)+ I(0)+ R(0)=N, \forall t. \end{aligned}$$

Thus, $\Gamma$ is positively invariant.

## Model validation

**Figure 2:**
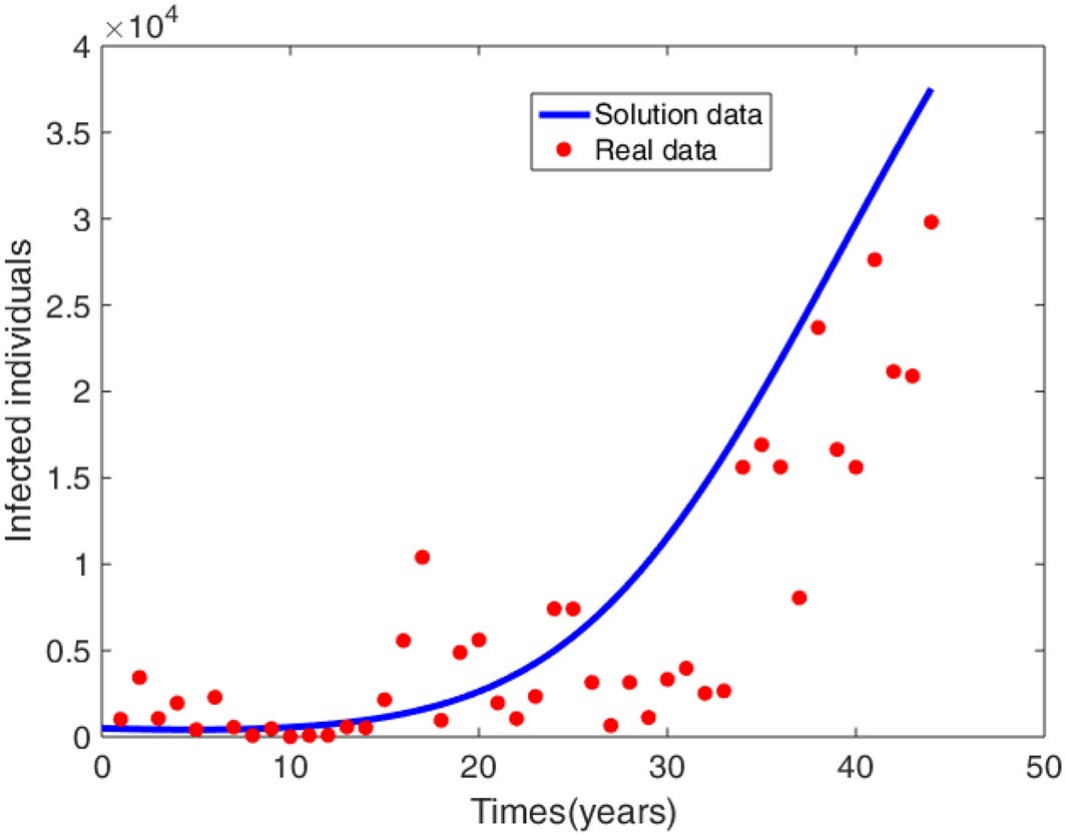
Real data (see Table 1) fitted with a model solution.

The importance of the developed mathematical model is validated against the WHO cholera data for Bangladesh recorded from 1950 to 2000^29^. The model solution of infected individuals is compared with the real data of number of reported cases of cholera, see Fig. 2 and Table 1. The difference between the real data and the model solution is calculated by the relative error (RE) formula given as^31,32^

17 $$\begin{aligned} RE= \frac{||I-\tilde{I} ||}{||\tilde{I}||}, \end{aligned}$$

where *I* is model solution for the infected individuals, $\tilde{I}$ is the real data of infected individuals and $||\times ||$ is a vector norm . Thus, it is calculated that $RE=0.5365$ which indicates a good fit between the model solution and the real data, see Fig. 2.

**Table 1:** WHO cholera data for Bangladesh, see^29^.

Year	Case number	Death number	CFR	Year	Case number	Death number	CFR
2000	1021	16	1.57	1972	1059	201	18.98
1999	3440	63	1.83	1971	2342	386	16.48
1998	1067	26	2.44	1970	7419	1889	25.46
1997	1959	95	4.85	1969	7411	1556	21
1996	418	0	0	1968	3156	614	19.46
1995	2297	61	2.66	1967	664	369	55.57
1994	562	41	7.3	1966	3154	1234	39.12
1993	78	0	0	1965	1123	683	68.82
1992	479	29	6.05	1964	3333	2419	72.58
1991	8	0	0	1963	3975	1248	31.4
1990	82	4	4.88	1962	2524	1304	51.66
1989	94	2	2.13	1961	2663	1703	63.95
1988	571	43	7.53	1960	15618	6272	40.16
1987	523	23	4.4	1959	16915	11056	65.36
1979	2154	21	0.97	1958	15631	10119	64.74
1978	5576	81	1.45	1957	8054	5134	63.74
1977	10403	354	3.4	1956	23699	15310	64.6
1976	957	62	6.48	1955	16642	9802	58.9
1975	4888	117	2.39	1954	15617	9443	60.47
1974	5614	173	3.08	1953	27631	16904	61.18
1973	1969	369	18.74	1952	21154	12884	60.91
1951	20894	12372	59.21	1950	29809	12947	43.43

## Mathematical analysis of equilibrium solutions

In this Section, the general properties of equilibrium solutions to the dynamical system in Eqs. (6)–(8) are presented.

### General properties of epidemic cholera dynamics

Equations (6)–(8) in the model provide a disease-free state as follows:

18 $$\begin{aligned} E_0 = (S^*,I^*,B^*) =\left( \frac{\mu N}{\mu +v},0,0 \right) . \end{aligned}$$

For the derivation of the basic reproduction number($\Re _0$), we have followed the procedures presented in^20,33,34^. We considered the infective compartments to be *I* and *B* so that the infective subsystem of the mathematical model to be

19 $$\begin{aligned} \frac{dI}{dt}= & {} (\alpha I + \beta B)S-\mu I- (\delta +a)I, \end{aligned}$$

20 $$\begin{aligned} \frac{dB}{dt}= & {} \xi I + r B\left( 1-\frac{B}{\kappa }\right) -\gamma B-wB. \end{aligned}$$

The linearized system of the Eqs. (19)–(20) about the DFE is given as

21 $$\begin{aligned} \frac{dI}{dt}= & {} \frac{\alpha \mu N}{\mu +v}I + \frac{\beta \mu N}{\mu +v} B-(\mu + \delta +a)I, \end{aligned}$$

22 $$\begin{aligned} \frac{dB}{dt}= {} \xi I + (r-\gamma -w)B. \end{aligned}$$

From Eqs. (21)–(22), the matrix of transmissions and matrix of transsission are, respectively given as

23 $$\begin{aligned} T=\begin{bmatrix} \alpha \frac{\mu N}{\mu +v} &{} \beta \frac{\mu N}{\mu +v} \\ 0 &{} 0 \end{bmatrix}\quad \text {and}\quad \, \Sigma =\begin{bmatrix} -(\mu +\delta +a) &{} 0 \\ \xi &{} r-(w+\gamma ) \end{bmatrix} . \end{aligned}$$

The basic reproduction number is the spectral radius of $-T\Sigma ^{-1}$. The eigenvalues of $-T\Sigma ^{-1}$ are

24 $$\begin{aligned} \lambda _1 = 0,\,\, \lambda _2=\frac{\mu N\left( \alpha (r-w-\gamma )-\beta \xi \right) }{(r-\gamma -w)(\mu +v)(\mu +\delta +a)}. \end{aligned}$$

Therefore, the basic reproduction number is

25 $$\begin{aligned} \Re _0=\frac{\mu N\left( \alpha (r-w-\gamma )-\beta \xi \right) }{(r-\gamma -w)(\mu +v)(\mu +\delta +a)}, \end{aligned}$$

provided that $r-w-\gamma <\frac{\beta \xi }{\alpha }$. Moreover, the expression $r-w-\gamma < 0$ must hold. Epidemiologically, this relation is meant the bacterium growth rate is lesser than the sum of the natural death rate of the bacterium and sanitation rate. The equation for the basic reproduction number’s denominator demonstrates how heavily the values of the control factors affect it. The fundamental reproduction number’s weight can be decreased by raising the control parameter settings.

#### Theorem 1

(Local equilibrium point stability in the absence of illness) The dynamical structure in Eqs. (6)–(8) has an unaffected by illness equilibrium point $E_0$ that is locally asymptotically stable if $\Re _0 <1$, and unstable if $\Re _0 >1$.

#### Proof

Theorem 2 in^33^ indicates that when $\Re _0 <1$, the disease-free equilibrium is locally asymptotically stable. On the other hand, if the controls are insufficiently robust to ensure that $\Re _0 >1$, the disease breakout happens and the DFE becomes unstable.

#### Theorem 2

(Global stability of the equilibrium point devoid of illness) The dynamical system in Eqs. (6)–(8) has a cholera-free equilibrium point $E_0$ that is globally asymptotically stable if $\Re _0 <1$, and unstable if $\Re _0 >1$.

#### Proof

To demonstrate that the disease-free equilibrium $E_0$ is globally asymptotically stable for $\Re _0 <1$ and unstable for $\Re _0 >1$, we will employ the LaSalle invariance principle^30,35^. Let $L(t):=B(t)$ be a Lyapunov function defined. The following Eqs. are thus valid.

26 $$\begin{aligned} \frac{dL}{dt}= & {} \frac{dB}{dt}=\xi I + r B\left( 1-\frac{B}{\kappa }\right) -\gamma B-wB \le \xi I + (r-(\gamma +w) )B. \end{aligned}$$

Setting $\frac{dI}{dt}=0$ in Eq. (7), we have that

27 $$\begin{aligned} I=\frac{\beta BS}{\mu +\delta +a -\alpha S}. \end{aligned}$$

Substituting Eq. (27) into Eq. (26), we have that

28 $$\begin{aligned} \frac{dL}{dt}\le \left( \frac{\xi \beta S}{\mu + \delta +a -\alpha S} + (r-(\gamma +w) \right) B. \end{aligned}$$

Evaluating Eq. (28) at $S=\frac{\mu N}{\mu +v}$, we obtained the following inequality.

29 $$\begin{aligned} \frac{dL}{dt}\le \frac{(\mu +\delta +a)(\mu +v)(r-\gamma -w)B}{\alpha \mu \left( N-\frac{(\mu +\delta +a)(\mu +v)}{\alpha \mu }\right) }\left( \Re _0-1\right) . \end{aligned}$$

From the above equation, the function $\frac{dL}{dt}$ is negative semi-definite for $\Re _0 \le 1$ provided that $N<\frac{(\mu +\delta +a)(\mu +v)}{\alpha \mu }$. From the definition of $\Re _0$, we saw that $r-\gamma -w <0$. Therefore, the largest compact invariant set in $\Gamma$ such that $\frac{dL}{dt} =0$ whenever $\Re _0 < 1$ is the singleton disease-free equilibrium. Thus, the global asymptotically stability of the disease-free equilibrium in $\Gamma$ is guaranteed by the LaSalle invariance principle^35^ whenever $\Re _0 \le 1$ and globally unstable for $\Re _0 > 1$ .

### General properties of endemic cholera dynamics

As previously stated, the DFE becomes unsustainable and the sickness will persist if the effects of the constraints are insufficient to bring $\Re _0$ below unity. Now let’s examine the endemic balance in order to understand the dynamics of cholera over the long run.

#### Theorem 3

(Existence of endemic equilibrium) If and only if $\Re _0>1$, there is a unique positive endemic equilibrium for the dynamical system in Eqs. (6)–(8).

#### Proof

The following nonlinear algebraic system has solutions, which are the endemic equilibrium solutions to the dynamical system in Eqs. (6)–(8).

30 $$\begin{aligned} \mu N= & {} \left( \alpha I + \beta B\right) S +\left( \mu + v\right) S, \end{aligned}$$

31 $$\begin{aligned} \left( \alpha I + \beta B\right) S= & {} \left( \mu + \delta +a\right) I, \end{aligned}$$

32 $$\begin{aligned} I= & {} \frac{r}{\xi \kappa } B^2 +\frac{\gamma +w-r}{\xi }B. \end{aligned}$$

After defining $\theta =\frac{\mu N}{\mu +\delta +a}$ and taking into account Eqs. (30)–(31), we obtain the following quadratic equation.

33 $$\begin{aligned} \alpha I^2 + \left[ \beta B +\mu +v -\alpha \theta \right] I -\beta \theta B=0. \end{aligned}$$

The function $I=f(B)$, defined as follows, is the answer to the problem above.

34 $$\begin{aligned} f(B)= \frac{\sqrt{\left( \mu +v-\alpha \theta +\beta B\right) ^2+4\alpha \beta \theta B }-\left( \mu +v-\alpha \theta +\beta B \right) }{2\alpha },\, B\geqslant 0. \end{aligned}$$

Once more, we create a function $B=h(I)$ from Eq. (33) in the following way.

35 $$\begin{aligned} h(I)= \frac{\left( \mu +v\right) I}{\beta \left( \theta -I \right) }-\frac{\alpha I}{\beta },\, I\geqslant 0. \end{aligned}$$

The following derivatives make it simple to verify that the functions *f* and *h* are connected.

36 $$\begin{aligned} f'\left( B\right) =\frac{1}{h'\left( I\right) }. \end{aligned}$$

#### Lemma 2

The fact that $h(I)=B>0$ implies the following relations.

(i) $$I< \theta $$,
(ii) $$\text {max}\left( 0, \theta -\frac{\mu +v}{\alpha }\right)<I<\theta $$.

#### Proof

The results in Lemma 2 are direct consequences of the inequality

37 $$\begin{aligned} \frac{\left( \mu +v\right) I}{\beta \left( \theta -I \right) }-\frac{\alpha I}{\beta }>0. \end{aligned}$$

We denote the expression in Eq. (32) as

38 $$\begin{aligned} I=g\left( B\right) = \frac{r}{\xi \kappa } B^2 +\frac{\gamma +w-r}{\xi }B. \end{aligned}$$

Thus, the existence of endemic equilibrium depends on the existence of solutions to the equation

39 $$\begin{aligned} f\left( B\right) =g\left( B\right) ,\, B>0. \end{aligned}$$

Assume that $n=\text {min}\left( \theta ,\frac{\mu +v}{\alpha }\right)$. Then, from the results of Lemma 2, we have that $0<\theta -I<n$ and $\left( \frac{\mu +v}{\alpha }\right) \theta \geqslant n^2$. This leads us to the following inequality.

40 $$\begin{aligned} \left( \mu +v \right) \theta -\alpha \left( \theta -I\right) ^2 > \left( \mu +v \right) \theta -\alpha n^2 =\alpha \left( \left( \frac{\mu +v}{\alpha }\right) \theta -n^2 \right) \geqslant 0. \end{aligned}$$

Considering the above equation and differentiating the functions *f* and *g*, we have

41 $$\begin{aligned} f'\left( B\right)= & {} \frac{1}{h'\left( I \right) }=\frac{\beta \left( \theta -I \right) ^2 }{\left( \mu +v \right) \theta -\alpha \left( \theta -I\right) ^2 }>0, \end{aligned}$$

42 $$\begin{aligned} f''\left( B\right)= & {} -\frac{h''\left( I\right) }{h'\left( I\right) ^3}=-\frac{2\left( \mu +v\right) \theta f'\left( B\right) ^3 }{\beta \left( \theta -I\right) ^3 }<0, \end{aligned}$$

43 $$\begin{aligned} g''\left( B\right)= & {} \frac{2r}{\xi \kappa }. \end{aligned}$$

The derivatives presented in the above equations are very important in examining the behavior of the function *f* and *g*. Evaluating $f'$ and $g'$ at $B=0$, we have

44 $$\begin{aligned} f'\left( 0\right)= & {} \frac{\beta \mu N}{\left( \mu +v \right) \left( \mu +\delta +a \right) }\left[ \frac{1}{1- \frac{\alpha \mu N}{\left( \mu +v \right) \left( \mu +\delta +a \right) }} \right] , \end{aligned}$$

45 $$\begin{aligned} g'\left( 0\right)= & {} \frac{\gamma +w-r}{\xi } . \end{aligned}$$

A direct consequence of the above equations is

46 $$\begin{aligned} f'\left( 0\right) -g'\left( 0\right) =\frac{\left( \gamma +w-r\right) \left( \Re _0-1 \right) }{\xi \left( 1-\frac{\alpha \mu N}{\left( \mu +v \right) \left( \mu +\delta +a \right) } \right) }. \end{aligned}$$

Now, lets consider the following case to analyze the existence of solution to the equation $f\left( B\right) =g\left( B\right)$.

Case I: $f\left( 0\right) >g\left( 0\right)$. In this case, it is clear that $g\left( 0\right) =0$. Therefore, $f\left( 0\right) >g\left( 0\right)$ implies that $\frac{\alpha \mu N}{\left( \mu +v \right) \left( \mu +\delta +a \right) } >1$ which means that $I>0$. Thus, since *f* is concave downward and *g* is concave upward, there exists a unique nontrivial equilibrium solution in this case.
Case II: $f\left( 0\right) =g\left( 0\right)$. In this case, the relation $\frac{\alpha \mu N}{\left( \mu +v \right) \left( \mu +\delta +a \right) } \leqslant 1$ holds. Thus, the existence depends on the relative slopes of *f* and *g* at $B=0$.
  (i) Consider the case $\frac{\alpha \mu N}{\left( \mu +v \right) \left( \mu +\delta +a \right) }=1$. From Eqs. (44)–(45), we have that
    47 $$\begin{aligned} f'\left( 0_+\right) =+\infty >g'\left( 0\right) . \end{aligned}$$
    Therefore, since *f* is concave downward and *g* is concave upward, there exists a unique nontrivial equilibrium solution in this case.
  (ii) Consider the case $\frac{\alpha \mu N}{\left( \mu +v \right) \left( \mu +\delta +a \right) }<1$. From the expression in Eq. (46), The next two cases are the ones that follow.
    Case (a): $f'\left( 0\right) >g'\left( 0\right)$. This is meant that $\Re _0 >1$. In this case, there exists a unique nontrivial equilibrium solution as *f* is concave downward and *g* is concave upward.
    Case (b): $f'\left( 0\right) <g'\left( 0\right)$. This is meant that $\Re _0 <1$. In this case, there doe not exist a nontrivial equilibrium solution as *f* is concave downward and *g* is concave upward.

Accordingly, the dynamical system in Eqs. (6)–(8) has a distinctive positive endemic equilibrium if and only if $\Re _0>1$.

#### Theorem 4

(Local stability of endemic equilibrium) The dynamical system in Eqs. (2)–(5) is locally asymptotically stable in its endemic equilibrium.

#### Proof

Assume that $EE= \left( S_1,I_1,R_1,B_1\right)$ represents the endemic equilibrium of the dynamical system in Eqs. (2)–(5). The dynamical system’s Jacobian matrix (*J*) at *EE* is provided as

48 $$\begin{aligned} J = \begin{bmatrix} -(\alpha I_1 +\beta B_1 +\mu +v) &{} -\alpha S_1&{}0&{}-\beta S_1 \\ \alpha I_1 +\beta B_1 &{} \alpha S_1 -(\mu +\delta +a)&{}0&{}\beta S_1\\ v&{}\delta +a&{}-\mu &{}0\\ 0&{}\xi &{}0&{}-\frac{r\left( 2B_1-\kappa \right) }{\kappa }-(\gamma +w) \end{bmatrix} . \end{aligned}$$

The Jacobian matrix’s characteristic polynomial is provided as

49 $$\begin{aligned} \left| \lambda I_4 -J\right| =\left( \lambda +\mu \right) \left( \lambda ^3 +a_1 \lambda ^2 +a_2 \lambda +a_3\right) , \end{aligned}$$

where $a_1$, $a_2$ and $a_3$ are given as in the following.

$$\begin{aligned} a_1= & {} \frac{\mu N}{S_1} + \frac{\beta B_1 S_1}{I_1} +\frac{\xi I_1}{B_1}+\frac{rB_1}{\kappa }, \\ a_2= & {} \frac{\mu N}{S_1}\left( \frac{\xi I_1}{B_1}+\frac{rB_1}{\kappa }\right) +\frac{\beta \mu NB_1}{I_1} + \alpha \left( \mu +\delta +a \right) I_1 + \frac{\beta rS_1B_1^2}{\kappa I_1},\\ a_3= & {} \frac{\beta r\mu N B_1^2}{\kappa I_1} + \alpha \left( \mu +\delta +a \right) I_1 \left( \frac{\xi I_1}{B_1}+\frac{rB_1}{\kappa }\right) +\beta \xi \left( \mu +\delta +a \right) I_1. \end{aligned}$$

For an endemic equilibrium $EE= \left( S_1,I_1,R_1,B_1\right)$, we have that $a_1>0$, $a_2>0$ and $a_3>0$. Moreover,

$$\begin{aligned} a_1 a_2> & {} \left( \frac{\mu N}{S_1} +\frac{\xi I_1}{B_1}+\frac{rB_1}{\kappa }\right) \left( \frac{\beta \mu NB_1}{I_1} + \alpha \left( \mu +\delta +a \right) I_1 + \frac{\beta rS_1B_1^2}{\kappa I_1}\right) ,\\> & {} \left( \frac{\mu N}{S_1}\right) \left( \frac{\beta rS_1B_1^2}{\kappa I_1}\right) + \left( \frac{\xi I_1}{B_1}+\frac{rB_1}{\kappa }\right) \left( \frac{\beta \mu NB_1}{I_1} + \alpha \left( \mu +\delta +a \right) I_1 \right) ,\\> & {} \frac{\beta r\mu N B_1^2}{\kappa I_1}+ \alpha \left( \mu +\delta +a \right) I_1 \left( \frac{\xi I_1}{B_1}+\frac{rB_1}{\kappa }\right) +\left( \frac{\xi I_1}{B_1} \right) \left( \frac{\beta \mu NB_1}{I_1} \right) ,\\> & {} \frac{\beta r\mu N B_1^2}{\kappa I_1}+ \alpha \left( \mu +\delta +a \right) I_1 \left( \frac{\xi I_1}{B_1}+\frac{rB_1}{\kappa }\right) +\beta \xi \left( \mu +\delta +a \right) I_1,\\= & {} a_3. \end{aligned}$$

The endemic equilibrium $EE= \left( S_1,I_1,R_1,B_1\right)$ is locally asymptotically stable, as per the Routh-Hurwitz criteria.

#### Theorem 5

(Global stability of endemic equilibrium) If $N\leqslant \frac{\delta +a-v}{2\alpha }$ holds, the dynamical system in Eqs. (2)–(5) is in an endemic equilibrium that is globally stable.

#### Proof

We apply the geometric approach proposed by Li and Muldowney^36^ to prove the global stability of endemic equilibrium $EE= \left( S,I,R,B\right)$. For simplicity, we drop the rate of change of the recovered individuals. Thus, the Jacobian matrix of the dynamical system is given as

$$\begin{aligned} J = \begin{bmatrix} -(\alpha I +\beta B +\mu +v) &{} -\alpha S&{}-\beta S \\ \alpha I +\beta B &{} \alpha S -(\mu +\delta +a)&{}\beta S\\ 0&{}\xi &{}-\frac{r\left( 2B-\kappa \right) }{\kappa }-(\gamma +w) \end{bmatrix} . \end{aligned}$$

Denoting $d=\frac{r\left( 2B_1-\kappa \right) }{\kappa }+(\gamma +w)$ and defining $A\left( t \right) =I_3 +t J$, the second additive compound matrix of *J* is given as $\Delta _2 \left( J \right) = \frac{d}{dt}\left( C_2\left( A(t)\right) \right) |_{t=0}$ where $C_2\left( A(t)\right)$ is the second compound matrix of $A\left( t \right)$, $I_3$ is the $3\times 3$ identity matrix and *t* is a scalar. Thus,

$$\begin{aligned} \Delta _2\left( J \right) = \begin{bmatrix} \alpha S-(\alpha I +\beta B +2\mu +v +\delta +a) &{} \beta S&{}\beta S \\ \xi &{} -\left( \alpha I +\beta B + \mu +v+d\right) &{}-\alpha S\\ 0&{}\alpha I +\beta B &{}\alpha S-\left( \mu +\delta +a+d\right) \end{bmatrix} . \end{aligned}$$

Define $P=\text {diag}\left[ 1,\frac{I}{B},\frac{I}{B}\right]$ and let *f* denote the vector field of the dynamical system. Moreover, define $P_f$ to be the derivative of *P* along the direction of *f*. Then

$$\begin{aligned} P_fP^{-1}=\text {diag}\left[ 0,\frac{\dot{I}}{I}-\frac{\dot{B}}{B},\frac{\dot{I}}{I}-\frac{\dot{B}}{B}\right] , \end{aligned}$$

and

$$\begin{aligned} P \Delta _2\left( J \right) P^{-1} = \begin{bmatrix} \alpha S-(\alpha I +\beta B +2\left( \mu +v\right) +\delta +a) &{} \beta S\frac{B}{I}&{}\beta S \frac{B}{I}\\ \frac{I}{B}\xi &{} -\left( \alpha I +\beta B + \mu +v+d\right) &{}-\alpha S\\ 0&{}\alpha I +\beta B &{}\alpha S-\left( \mu +\delta +a+d\right) \end{bmatrix} . \end{aligned}$$

Now, we define a matrix $$Q:=P_fP^{-1}+P C_2\left( J \right) P^{-1}= \begin{bmatrix} Q_{11} &{} Q_{12} \\ Q_{21} &{} Q_{22} \end{bmatrix}$$ where there block matrices are given as

$$\begin{aligned} Q_{11}= & {} \alpha S-(\alpha I +\beta B +2\mu +v +\delta +a),\, Q_{12}=\left[ \beta S\frac{B}{I},\beta S\frac{B}{I}\right] ,\, Q_{21}=\left[ \xi \frac{I}{B},0\right] ^T,\\ Q_{22}= & {} \begin{bmatrix} -(\alpha I +\beta B +\mu +v +d) + \frac{\dot{I}}{I}-\frac{\dot{B}}{B}&{} -\alpha S\\ \alpha I +\beta B &{} \alpha S-\left( \mu +\delta +a+d\right) +\frac{\dot{I}}{I}-\frac{\dot{B}}{B} \end{bmatrix}. \end{aligned}$$

Let the Lozinski measure with respect to $L_\infty$ vector norm be denoted by *m*. Then, by a direct calculation, we found that

50 $$\begin{aligned} m\left( Q\right) =\text {sup}\left( g_1, g_2\right) , \end{aligned}$$

where

51 $$\begin{aligned} g_1= & {} m_1\left( Q_{11}\right) +\left| Q_{12}\right| =\alpha S +\beta S\frac{B}{I}-\left( \alpha I +\beta B +2\mu +v+\delta +a\right) ,\end{aligned}$$

52 $$\begin{aligned} g_2= & {} m_1\left( Q_{22}\right) +\left| Q_{21}\right| = \frac{\dot{I}}{I}-\mu -v-\frac{rB}{\kappa } + \text {sup}\left( 0, 2\alpha +v-\delta -a\right) . \end{aligned}$$

From the assumption $N\le \frac{\delta +a-v}{2\alpha }$, we have that $2\alpha S+v-\delta -a\le 0$ which implies that $\text {sup}\left( 0, 2\alpha +v-\delta -a\right) =0$. Moreover, from Equation (7), we have that $\beta S \frac{B}{I}=\frac{\dot{I}}{I}-\alpha S + \left( \mu +\delta +a\right)$. Thus, we have the following results.

53 $$\begin{aligned} g_1= & {} \frac{\dot{I}}{I}-\mu -\left( \alpha I+\beta B+v \right) \le \frac{\dot{I}}{I}-\mu , \end{aligned}$$

54 $$\begin{aligned} g_2= & {} \frac{\dot{I}}{I}-\mu -\left( v+\frac{rB}{\kappa }\right) \le \frac{\dot{I}}{I}-\mu , \end{aligned}$$

which implies that $m\left( Q\right) \le \frac{\dot{I}}{I}-\mu$. Since $0\le I(t)\le N$, there exists $T>0$ such that

55 $$\begin{aligned} \frac{\ln \left( I(t)\right) -\ln \left( I(0)\right) }{t}<\frac{\mu }{2}, \end{aligned}$$

for $t>T$. The above equation implies that

56 $$\begin{aligned} \frac{1}{t}\int ^{t}_{0}m\left( Q\right) d\tau \le \frac{1}{t}\int ^{t}_{0}\left( \frac{\dot{I}}{I}-\mu \right) d\tau = \frac{\ln \left( I(t)\right) -\ln \left( I(0)\right) }{t}-\mu \le -\frac{\mu }{2}. \end{aligned}$$

Thus, according to the geometric approach originally proposed by Li and Muldowney, the quantity $\bar{q}_2$ is given as

57 $$\begin{aligned} \bar{q}_2:=\limsup _{t\rightarrow \infty }\left( \frac{1}{t}\int ^{t}_{0}m\left( Q\right) d\tau \right) \le -\frac{\mu }{2}< 0. \end{aligned}$$

Therefore, the idea of geometric approach tells us that the endemic equilibrium is globally stable for $N\le \frac{\delta +a -v}{2\alpha }$.

## Numerical test problems for stability of equilibrium solutions

In all the numerical test problems, we consider $N=250000$ and $\kappa =10^6$.

### Numerical test problems for epidemic cholera dynamics

In this sub section, we are examining the results in Theorem 1 and 2 numerically for the epidemic cholera dynamics.

#### Example 1

We take into account the parameter values in Table 2 in this test problem.

With the parameter values in Table 2, $\Re _0 =0.000127412 <1$ and the only equilibrium solution to the dynamical system in Eqs. (6)–(8) is the disease free equilibrium given as

$$\begin{aligned} E_0 = \left( \frac{\mu N}{\mu +v},0,0 \right) =\left( 19.6535,0,0 \right) . \end{aligned}$$

The above disease free equilibrium is, hence, stable by Theorem 1 and 2.

**Figure 3:**
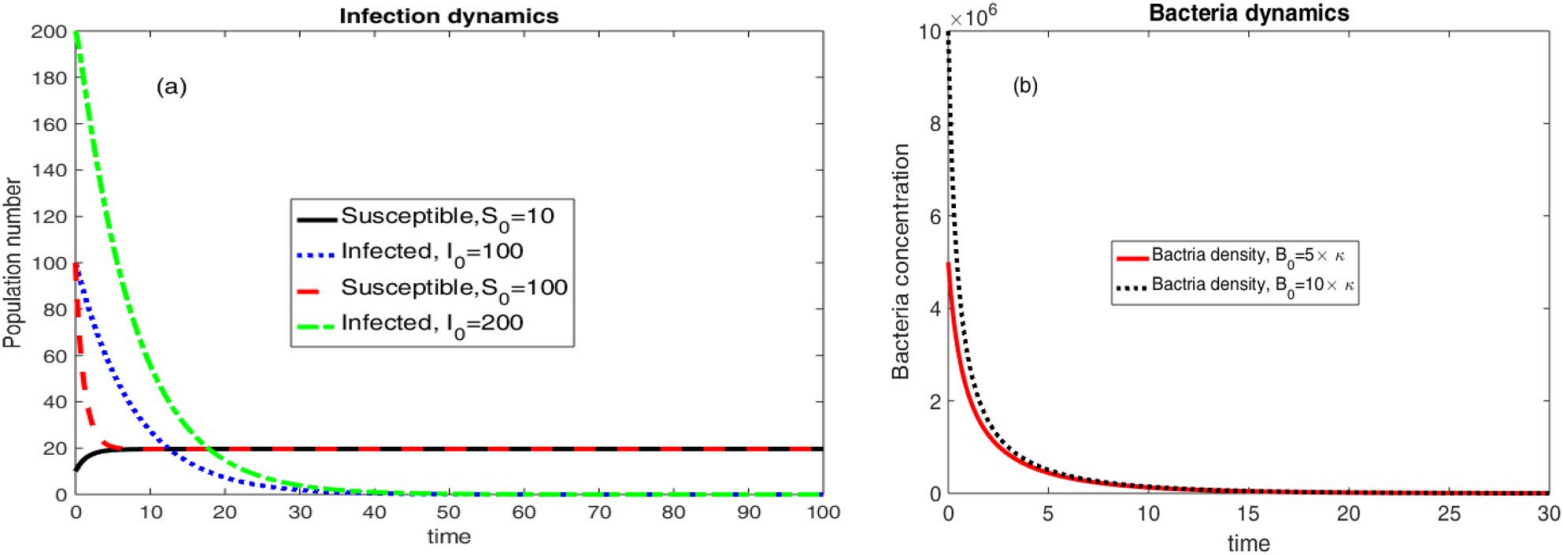
Population and Bacteria dynamics considering different initial conditions and $\Re _0 <1$.

Two different initial conditions $\left( S(0),I(0),B(0)\right) =\left( 10,100,5\times \kappa \right)$ and $\left( S(0),I(0),B(0)\right) =\left( 100,200,10\times \kappa \right)$ were considered to produce the results in Fig. 3. The results are eventually approaching to the disease free equilibrium.

**Table 2:** Parameter values in the case of 1.

Model parameter	Value	Reference	Model parameter	Value	Reference
$\alpha$	$1.48\times 10^{-8}$	^16^	$\beta$	$1.70\times 10^{-8}$	^16^
$\gamma$	$\frac{1}{10}$	^16,25^	$\delta$	$\frac{1}{30}$	^16,25^
$\xi$	10	^16,25^	*a*	0.1	assumed
*v*	0.8	assumed	*w*	0.3	assumed
*r*	0.2	assumed	$\mu$	1/43.5 year	^16,25^

**Table 3:** Parameter values for Example 2.

Model parameter	Value	Reference	Model parameter	Value	Reference
$\alpha$	0.0048	^16^	$\beta$	$1.70\times 10^{-8}$	^16^
$\gamma$	$\frac{1}{10}$	^16,25^	$\delta$	$\frac{1}{30}$	^16,25^
$\xi$	50	^16,25^	*a*	0.2	assumed
*v*	0.2	assumed	*w*	0.5	assumed
*r*	0.2	assumed	$\mu$	1/43.5 year	^16,25^

#### Example 2

In this test problem, we consider the values of parameters in Table 3.

Considering the values of parameters in Table 3, $\Re _0 =1.6171 >1$. In this case, we obtained two equilibrium solutions, Namely; the epidemic equilibrium and the endemic equilibrium for the dynamical system in Eqs. (6)–(8). These equilibrium solutions are given as

$$\begin{aligned} E_0 =\left( \frac{\mu N}{\mu +v},0,0 \right) =\left( 78.5953,0,0 \right) ,\, \text {and} \,\, E =\left( 48.6027,25.7091,3208.49 \right) . \end{aligned}$$

Thus, Theorems 1 and 2 tells us that the disease free equilibrium solution $E_0$ is unstable.

**Figure 4:**
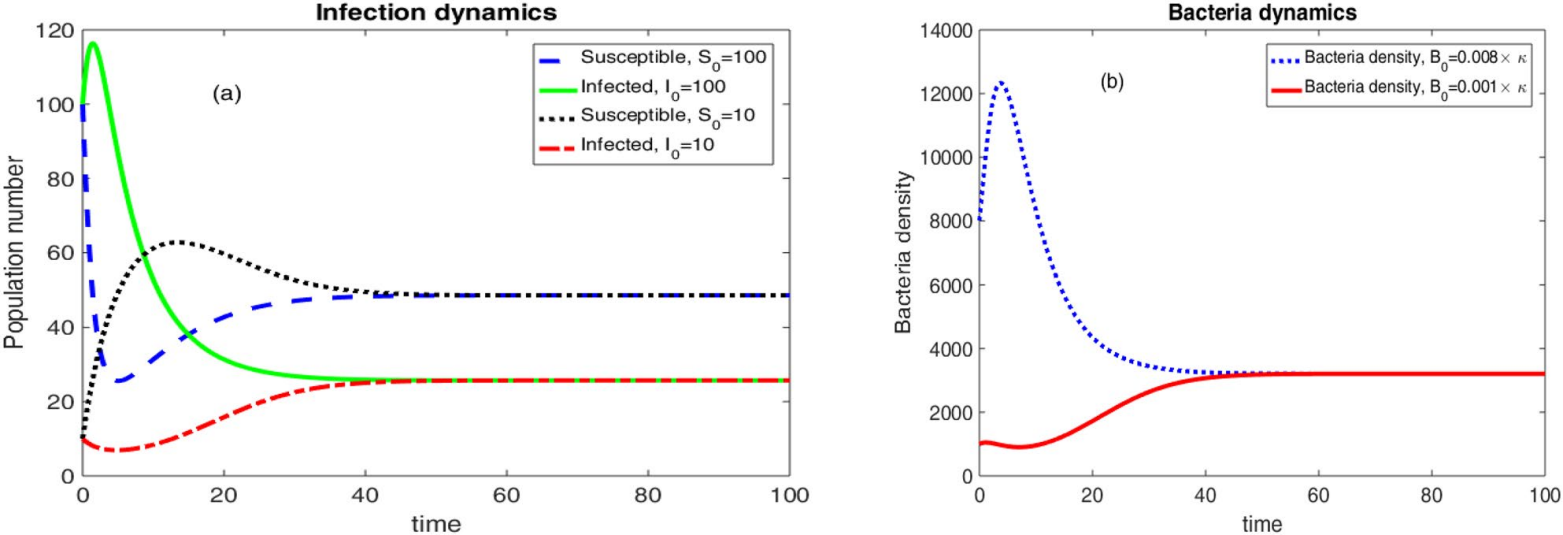
Population and Bacteria dynamics considering different initial conditions and $\Re _0 >1$.

Two different initial conditions $\left( S(0),I(0),B(0)\right) =\left( 100,100,0.008\times \kappa \right)$ and $\left( S(0),I(0),B(0)\right) =\left( 10,10,0.001\times \kappa \right)$ were considered to produce the results in Fig. 4. The results are eventually going away from the disease free equilibrium. Rather, the dynamical system is approaching to the endemic equilibrium.

### Numerical test problems for endemic cholera dynamics

In this sub section, we are examining the results in Theorem (3-5) numerically for the endemic cholera dynamics.

**Table 4:** Parameter values for Example 3.

Model parameter	Value	Reference	Model parameter	Value	Reference
$\alpha$	0.003	^16^	$\beta$	$1.70\times 10^{-8}$	^16^
$\gamma$	$\frac{1}{10}$	^16,25^	$\delta$	$\frac{1}{30}$	^16,25^
$\xi$	50	^16,25^	*a*	0.2	Assumed
*v*	0.2	Assumed	*w*	0.5	Assumed
*r*	0.2	Assumed	$\mu$	1/43.5 year	^16,25^

#### Example 3

In this test problem, we consider the values of parameters in Table 4.

**Figure 5:**
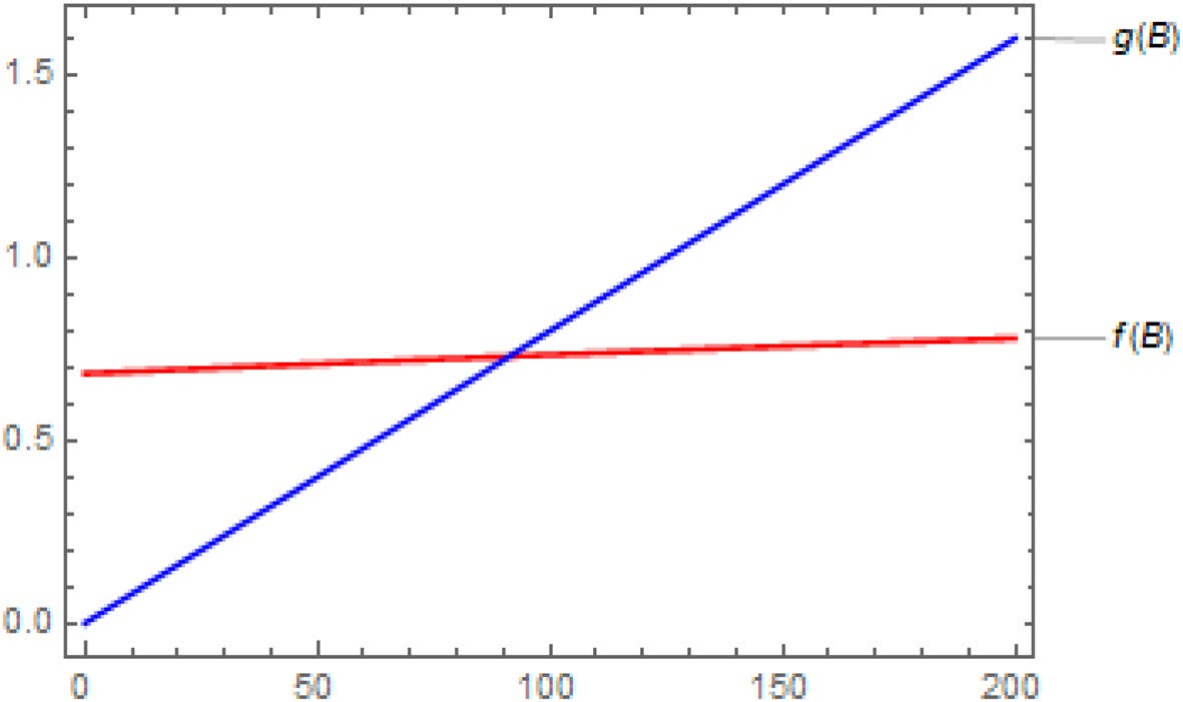
Comparison of $I=f\left( B\right)$ and $I=g\left( B\right)$ for endemic equilibrium.

Accordingly, $\frac{\alpha \mu N}{\left( \mu +v \right) \left( \mu +\delta +a \right) } = 1.01024>1$ and $\Re _0 =1.01095 >1$. This implies that $f\left( 0\right) > g\left( 0\right)$ which tells us a unique endemic equilibrium exists by Theorem 3. The existence of the endemic equilibrium is depicted in Fig. 5.

As can be seen from Fig. 5, the endemic equilibrium exists and is given as

$$\begin{aligned} E =\left( 77.7437, 0.730033, 91.25 \right) . \end{aligned}$$

Moreover, the epidemic equilibrium also exists and is given as

$$\begin{aligned} E_0 =\left( \frac{\mu N}{\mu +v},0,0 \right) =\left( 78.5953, 0,0\right) . \end{aligned}$$

**Figure 6:**
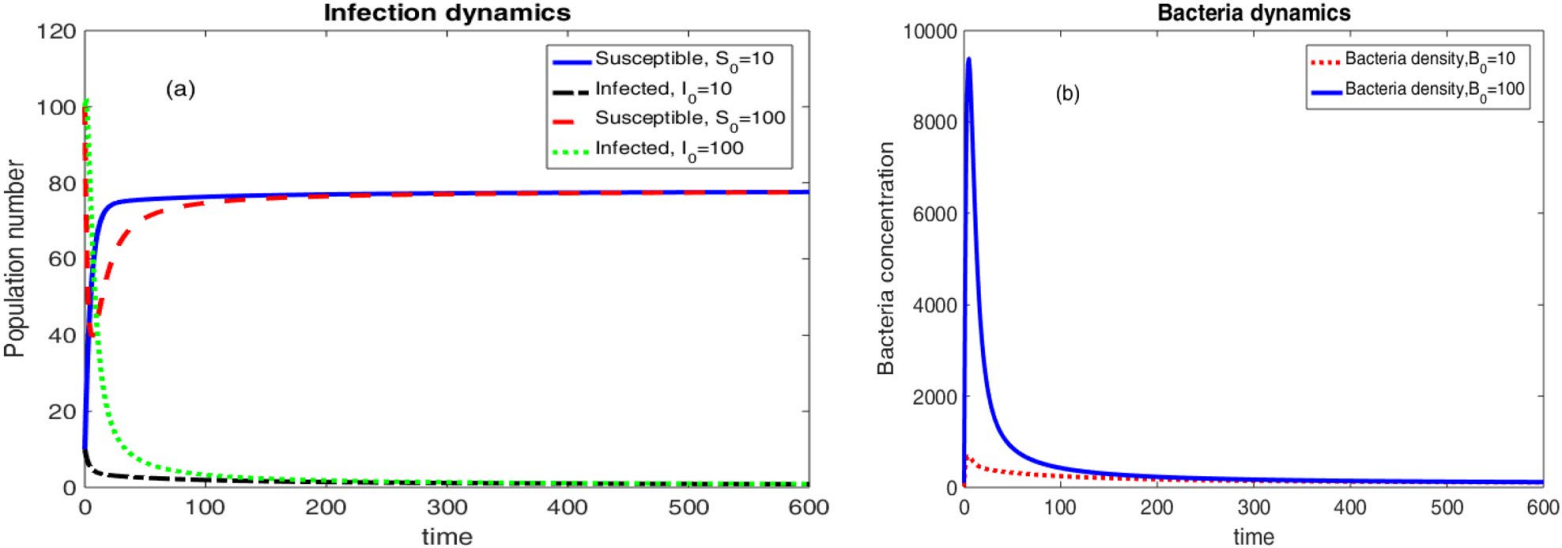
Endemic cholera dynamics.

Thus, Theorem 1 and Theorem 2 tells us that the disease free equilibrium solution $E_0$ is unstable. However, the endemic equilibrium $E =\left( 77.7437, 0.730033, 91.25 \right)$ is asymptotically stable by Theorem 4. We considered two different initial conditions $\left( S(0),I(0),B(0)\right) =\left( 10,10,10 \right)$ and $\left( S(0),I(0),B(0)\right) =\left( 100,100,100 \right)$ to produce the results in Fig. 6. It is shown that the dynamical system is approaching to the endemic equilibrium $E =\left( 77.7437, 0.730033,91.25 \right)$ asymptotically.

## Model parameter sensitivity analysis

The sensitivity of a model parameter (*p*) is meant its effect on the values of the basic reproduction number and is measured by the elasticity index defined as^37,38^

58 $$\begin{aligned} {\mathbb {Y}}_p^{\Re _0}= \frac{\partial \Re _0 }{\partial p}\times \frac{p}{\Re _0}. \end{aligned}$$

From the above equation, if the sign of ${\mathbb {Y}}_p^{\Re _0}$ is positive, the value of $\Re _0$ increases with an increase in the value of the model parameter. Moreover, if the sign of ${\mathbb {Y}}_p^{\Re _0}$ is negative, the value of $\Re _0$ decreases with an increase in the value of the model parameter^37^. The elasticity index is very important to guide an intervention by indicating the most important model parameters to target. Based on Equation (58), the elasticity index of the intervention strategies and intrinsic bacteria growth are derived to be

59 $$\begin{aligned} {\mathbb {Y}}_v^{\Re _0}= & {} -\frac{v}{\mu +v}<0, \end{aligned}$$

60 $$\begin{aligned} {\mathbb {Y}}_w^{\Re _0}= & {} -\frac{\beta \xi w}{\left( r-w-\gamma \right) \left( \alpha \left( r-w-\gamma \right) -\beta \xi \right) }<0, \end{aligned}$$

61 $$\begin{aligned} {\mathbb {Y}}_a^{\Re _0}= & {} -\frac{a}{\mu +\delta +a}<0, \end{aligned}$$

62 $$\begin{aligned} {\mathbb {Y}}_r^{\Re _0}= & {} \frac{\alpha \beta \xi r}{\left( r-w-\gamma \right) \left( \alpha \left( r-w-\gamma \right) -\beta \xi \right) }>0 \end{aligned}$$

From the theoretical point of view, intervention strategies play a great role in reducing the basic reproduction number and the intrinsic bacteria growth rate in increasing the basic reproduction number. The results in Eqs. (59)–(62) validates the theoretical studies on the effect of intervention strategies and intrinsic bacteria growth on basic reproduction numbers.

**Figure 7:**
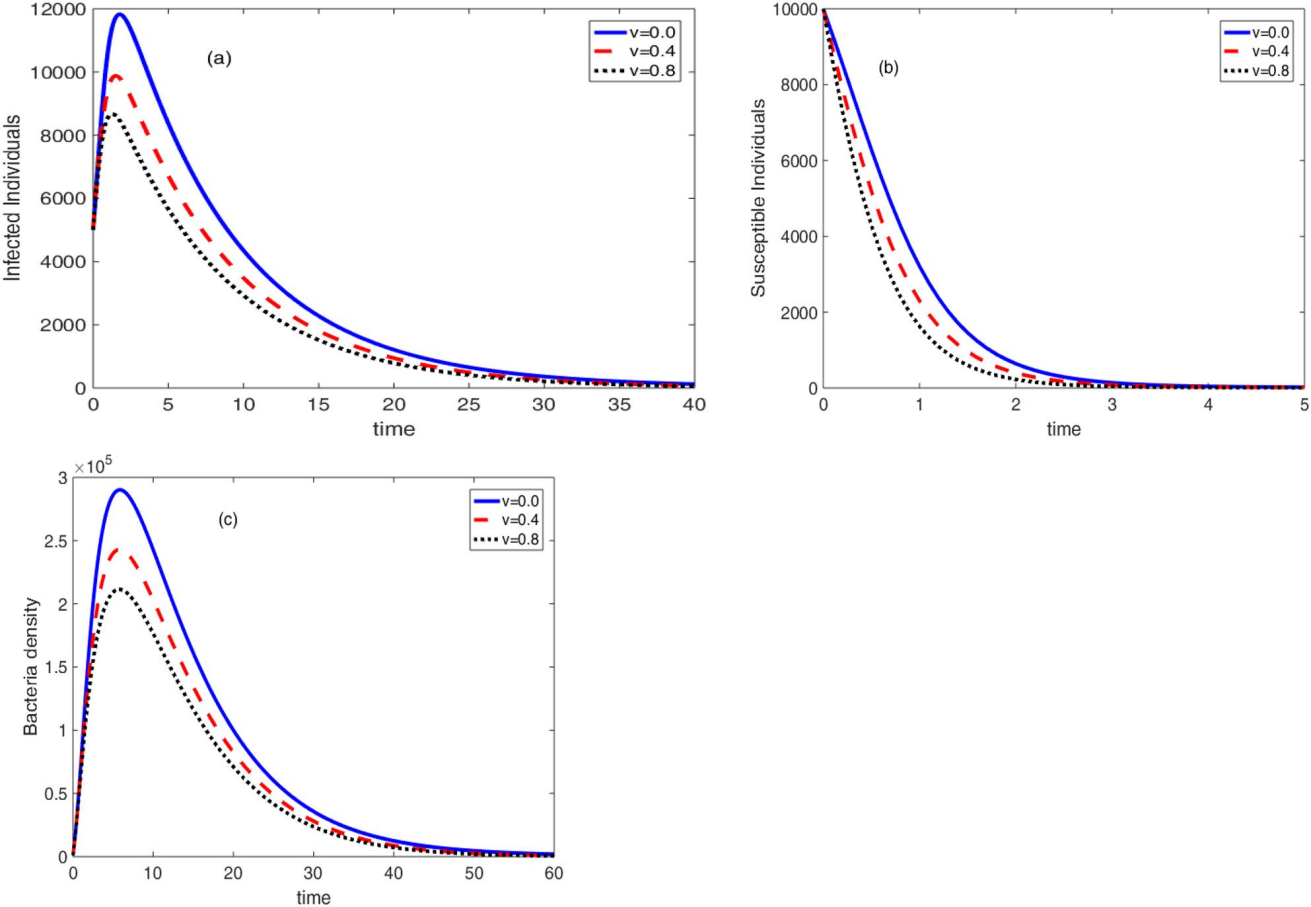
Effect of vaccine on the cholera dynamics.

**Figure 8:**
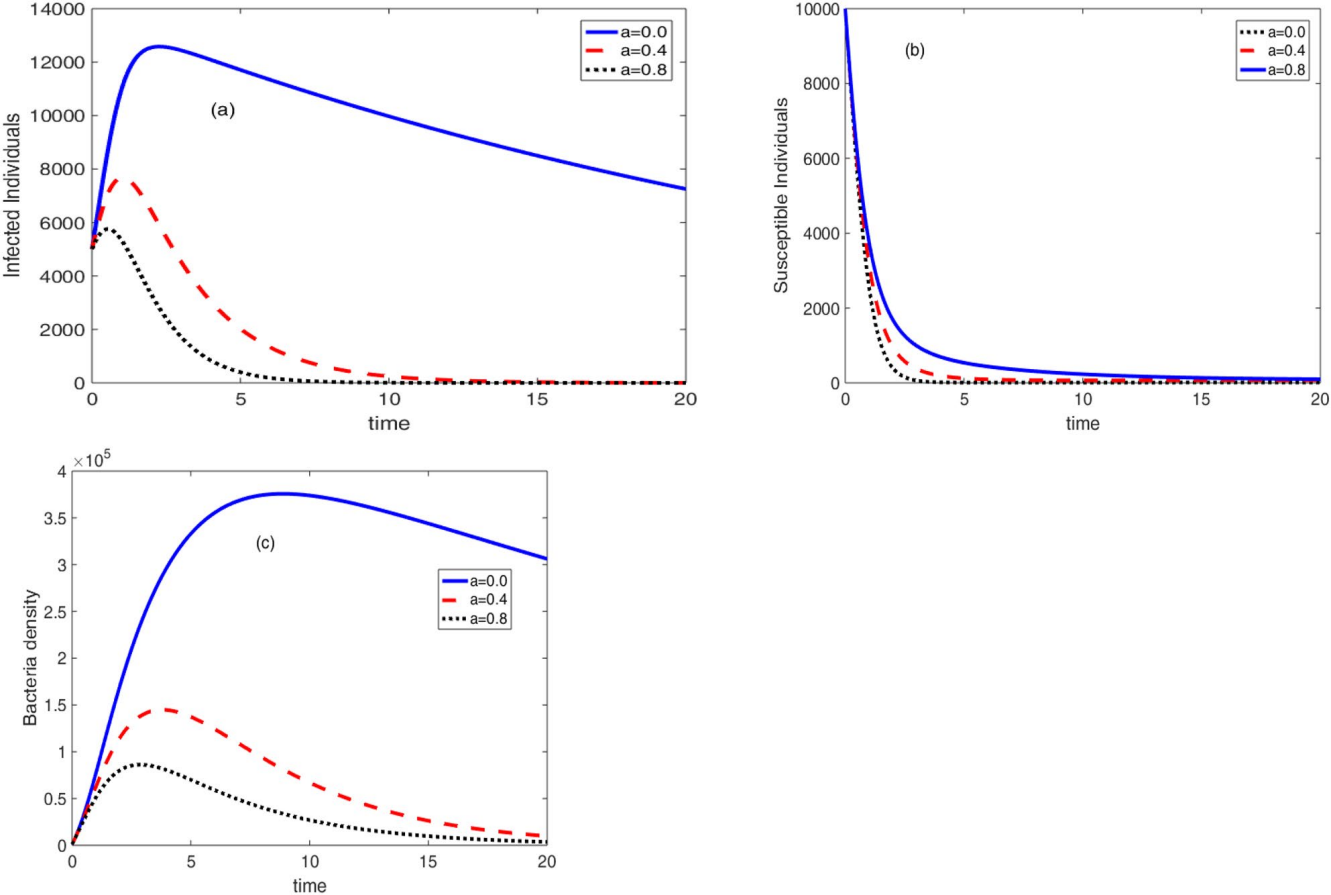
Effect of therapeutic treatment on the cholera dynamics.

**Figure 9:**
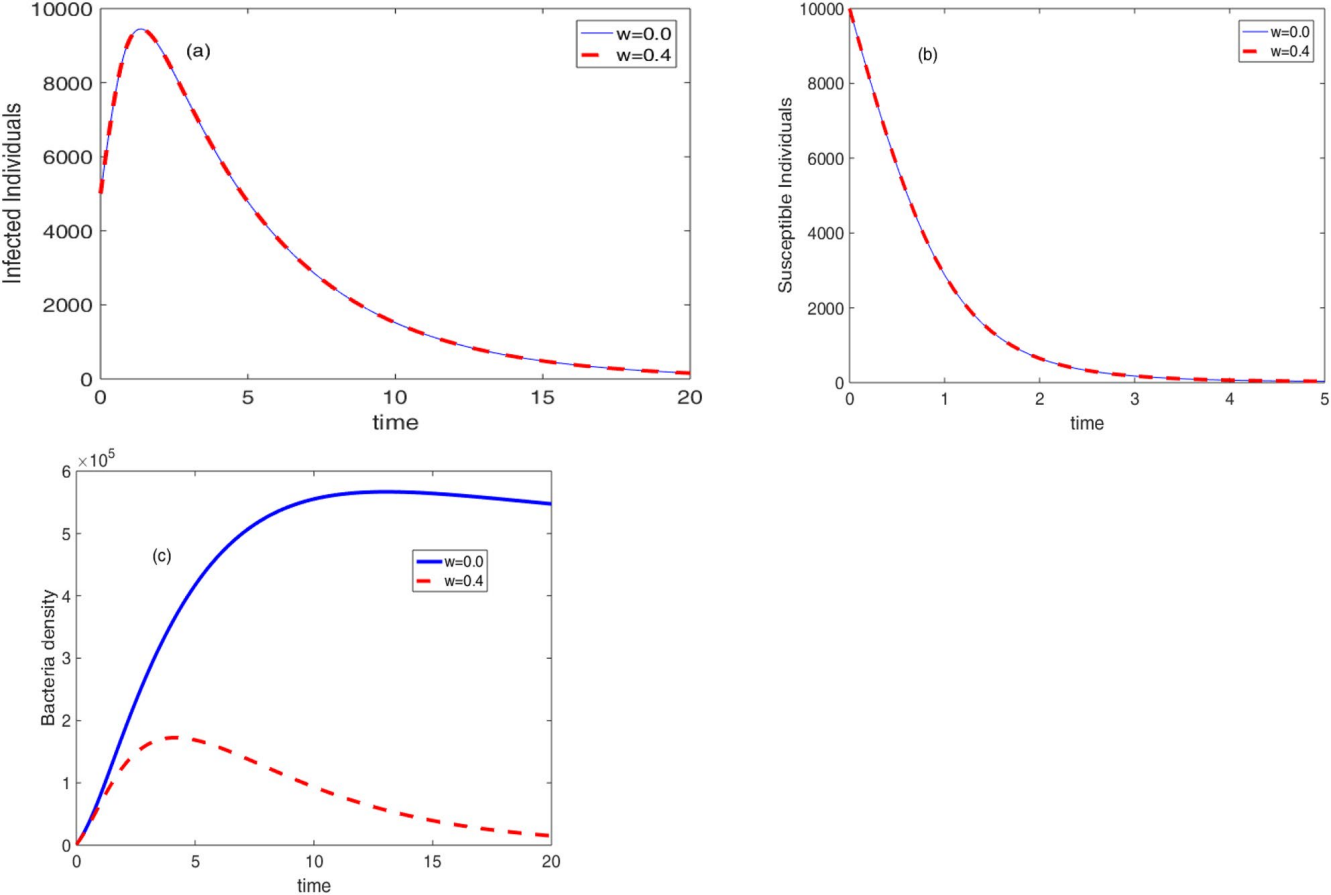
Effect of sanitation on the cholera dynamics.

Based on the values of the parameters in Table 2, the effects of vaccination, therapeutic treatment, and sanitation are shown in Figs. 7, 8 and 9. The impact of vaccination on the dynamical system is shown in Fig. 7. It is evident that a higher vaccination rate lowers the population of vulnerable people, the number of sick people, and the density of germs. As the vaccine is given to susceptible individuals, it drastically reduces the number of susceptible individuals within a short period of time as compared to the number of infected individuals and bacteria density.

Figure 8, the effect of therapeutic treatment is displayed. From the theoretical point of view, therapeutic treatment helps individuals to recover and they can not contribute bacteria to the environment. Because of this, when the rate of therapeutic treatment increases, so does the concentration of germs in the environment and the number of infected persons. The rate of therapeutic treatment and the rate of immunisation, however, are negatively correlated. This important result recommends the development for an optimal control problem. The values of the triplet control parameters $\left( v,a,w\right)$ that simultaneously reduce the number of susceptible, infective and bacteria density can be obtained using optimal control problem.

The effect of sanitation on the dynamics of the cholera disease is presented in Fig. 9. It shows that the bacteria concentration in the environment vanishes with an increase in the sanitation rate. However, in this test situation, there have been no appreciable changes in the number of susceptible and infected persons with an increase in sanitation rate. This suggests that in order to see a change, adjustments must be made to the remaining model parameter values. In other words, future research must examine the covariance of sanitation rate with the other model.

**Figure 10:**
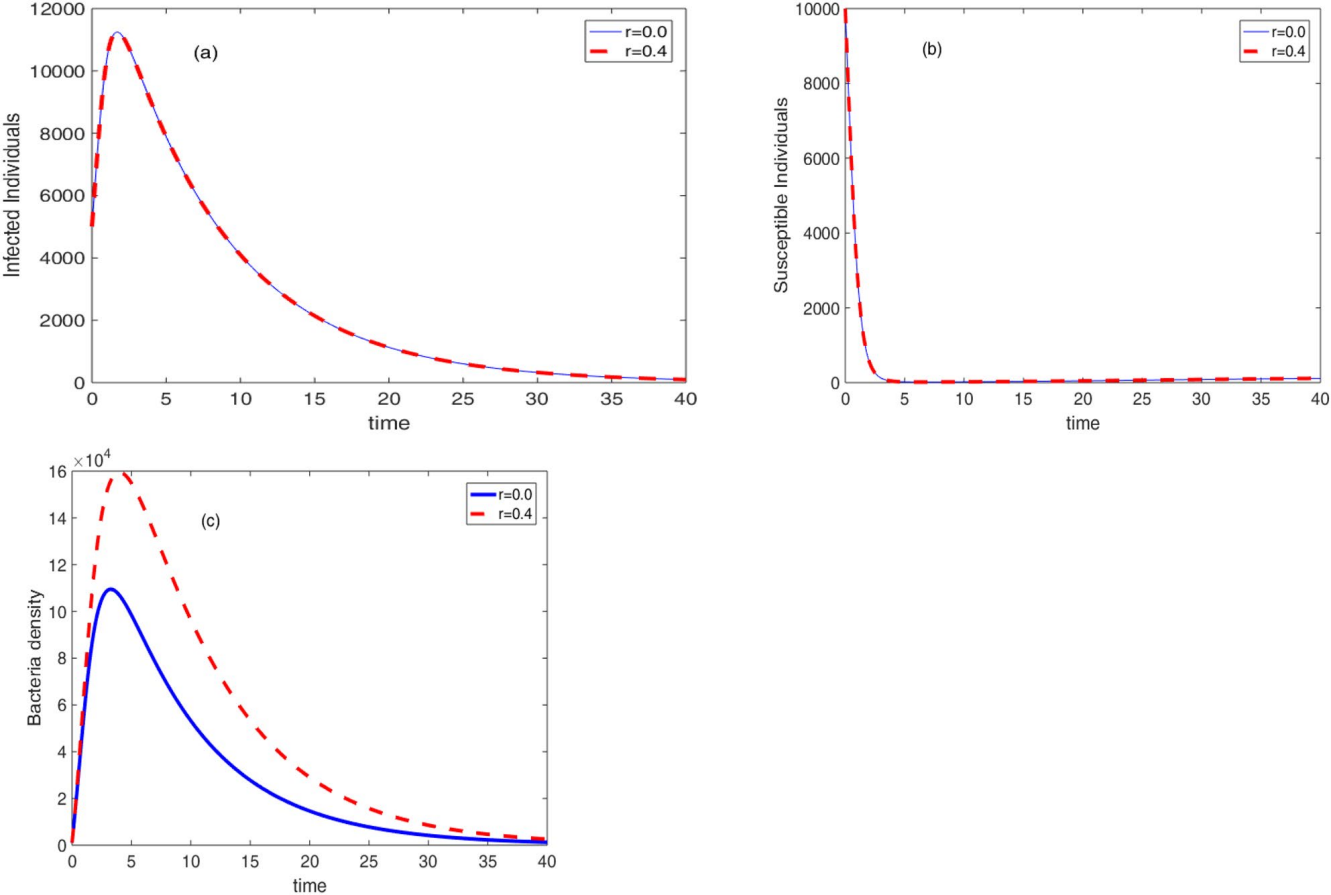
Effect of bacteria growth rate on the cholera dynamics.

The effect of bacteria growth rate on the infection dynamics is presented in Fig. 10. It can be observed that an increase in the growth rate results in an increase in the concentration of the bacteria. Moreover, it is displayed in Fig. 10b that the number of susceptible individuals starts to rise after decaying due to the growth rate.

## Conclusion and future works

In our assumption, we considered the bacteria intrinsic growth, vaccination, water sanitation and therapeutic treatment rate as very important parameters. The importance of these model parameters is embedded in the mathematical expression of the basic reproduction number. According to the results from sensitivity analysis, an increase in the bacteria intrinsic growth rate contributes positively to the value of the basic reproduction number. Nonetheless, it is determined that higher intervention rates have a detrimental impact on the fundamental reproduction number’s value. A significant discovery of this study is that a quick drop in infection may be achieved by maintaining a constant vaccination rate while adjusting treatment and sanitation rates.

The values of model parameters are obtained either from previous research works or are assumed by the researcher. In future works, the values of the model parameters can be estimated from real data using appropriate theories of approximations and covariance of model parameters has to be carried out to examine their relationship. Moreover, from an optimization point of view, intervention strategies should have to vary with time. Therefore, the formulation of an optimal control problem for the mathematical model is recommended in future works.

## Data Availability

The datasets generated and/or analysed during the current study are available from the corresponding author on reasonable request.
